# Anal fin pigmentation in *Brachyrhaphis* fishes is not used for sexual mimicry

**DOI:** 10.1371/journal.pone.0194121

**Published:** 2018-03-19

**Authors:** Kandace M. Flanary, Jerald B. Johnson

**Affiliations:** 1 Evolutionary Ecology Laboratories, Department of Biology, Brigham Young University, Provo, Utah, United States of America; 2 Monte L. Bean Life Science Museum, Brigham Young University, Provo, Utah, United States of America; Universidad Nacional Autonoma de Mexico, MEXICO

## Abstract

Mimicry can occur in several contexts, including sexual interactions. In some cases, males mimic females to gain access to potential mates. In contrast, there are relatively few examples of species where females mimic males, and we know very little about what drives these patterns. Two hypotheses have been advanced to explain female mimicry of males. The first is that mimicry is used to reduce harassment of females by males. The second is that mimicry is used to display dominance over other females. In this study, we tested these hypotheses in *Brachyrhaphis* fishes, wherein females of several species have pigmentation on their anal fin of the same coloration and shape, and in the same location, as the genitalia of males. To test if female mimicry of males reduces male harassment, we experimentally manipulated female pigmentation and observed male preference for females with and without male-like pigmentation. To test the effect that female mimicry of males has on female dominance, we observed how females respond to anal fin pigmentation patterns of companion females. We found that neither of these hypotheses was supported by our data. We conclude that similarities in anal fin pigmentation between male and female *Brachyrhaphis* fishes is not an adaptation to reduce male harassment or to signal dominance between females. Alternative explanations must exist, including the possibility that these similarities are simply non-adaptive.

## Introduction

Visual signals are used in many taxa as a way to communicate both within and among species [1, 2]. Success of the visual signal depends on the efficiency of the signal and the ability of the receiver to perceive and appropriately interpret the signal [3]. In some systems, individuals have evolved traits that mimic the signals of others. Mimicry can deter predators [4, 5], lure prey [6] or deceive hosts to allow the mimic to act as a parasite [7]. Mimicry can also occur in a sexual context. Sexual mimicry occurs when one sex mimics the other sex of the same species. Examples are found in a variety of taxa (fruit flies, [8]; cicadas, [9]; fishes,[10, 11]; hyenas, [12]; and flycatchers, [13]). In most cases of sexual mimicry, males mimic females. For example, in bluegill sunfish, small “sneaker” males deceive larger male bluegill sunfish into thinking the small males are in fact females. This allows for small males to enter into the nest and fertilize eggs that larger males would otherwise fertilize [14]. Similar examples of sneaker males are found in a variety of taxa (side-blotched lizards, [15]; goby fish, [11]; cuttlefish, [16]).

Sexual mimicry of males by females is uncommon. Moreover, there have been very few studies performed to explain why it may be adaptive for a female to mimic a male. Yet, two compelling hypotheses have been advanced. First, male mimicry could be used to reduce male harassment. For example, female damselflies are known to mimic males to avoid long, unwanted copulations [17, 18]. In this system, males are less attracted to females that look like males than to typical females. By reducing male harassment, females are able to dedicate more time and energy to obtaining nutrients needed for survival [19]. The second hypothesis is that male-mimicry could be used by females to establish dominance hierarchies. In other words, females might co-opt male traits and use them to signal their status to other females. This hypothesis stems from data in spotted hyenas, where females develop a pseudopenis that mimics a male penis. Pseudopenis size is positively related to female dominance status [20]. However, it remains unclear why mimicry of the male penis is used as a dominance signal in this system.

Fishes in the genus *Brachyrhaphis* appear to also show female sexual mimicry [21]. *Brachyrhaphis* are livebearers in the subfamily Poeciliinae, a monophyletic group of New World freshwater fishes characterized by internal female fertilization and external male genitalia (gonopodia) [22]. Male *Brachyrhaphis* fishes mate either by courting females or by attempting to force copulations when courtship is unsuccessful [23]. In *Brachyrhaphis*, females typically prefer the largest available males, while males typically pursue females closer to their own size [24]. Males either mate using courtship displays or by forced copulation, spending much of their time attempting to inseminate females [25]. What is most striking about *Brachyrhaphis* species is that females have melanin-based anal fin pigmentation, similar in shape, size, and color to gonopodial pigmentation of their male counterpart [21]. Furthermore, females display their anal fin to conspecifics in such a way as to clearly show their gonopodial-like anal fin pigmentation [21]. Interestingly, in *B*. *episcopi*, females show high levels of aggression when interacting with their own image via a mirror [26]. It is not clear why females show such aggressive displays, but it could be related to the dominance hypothesis described above. The convergence of gonopodial and anal fin pigmentation is found in all *Brachyrhaphis* species (12 total [27]), except *B*. *hartwegi* [21].

Hence, in this study we explored the two hypotheses that: (1) females anal fin pigmentation is present to reduce male attention; and (2) females anal fin pigmentation is used to signal dominance to other females. We predicted that males would be more attracted to unpigmented females than to females with the markings on their anal fin that mimicked the male gonopodium. We also predicted that females who interact with a pigmented female would show fewer aggressive behavioral displays than females who interact with a non-pigmented female. Surprisingly, we found that neither of these hypotheses was supported in *Brachyrhaphis* fishes, suggesting that the similarity in pigmentation between males and females may be non-adaptive.

## Materials and methods

### Study system

**Fig 1:**
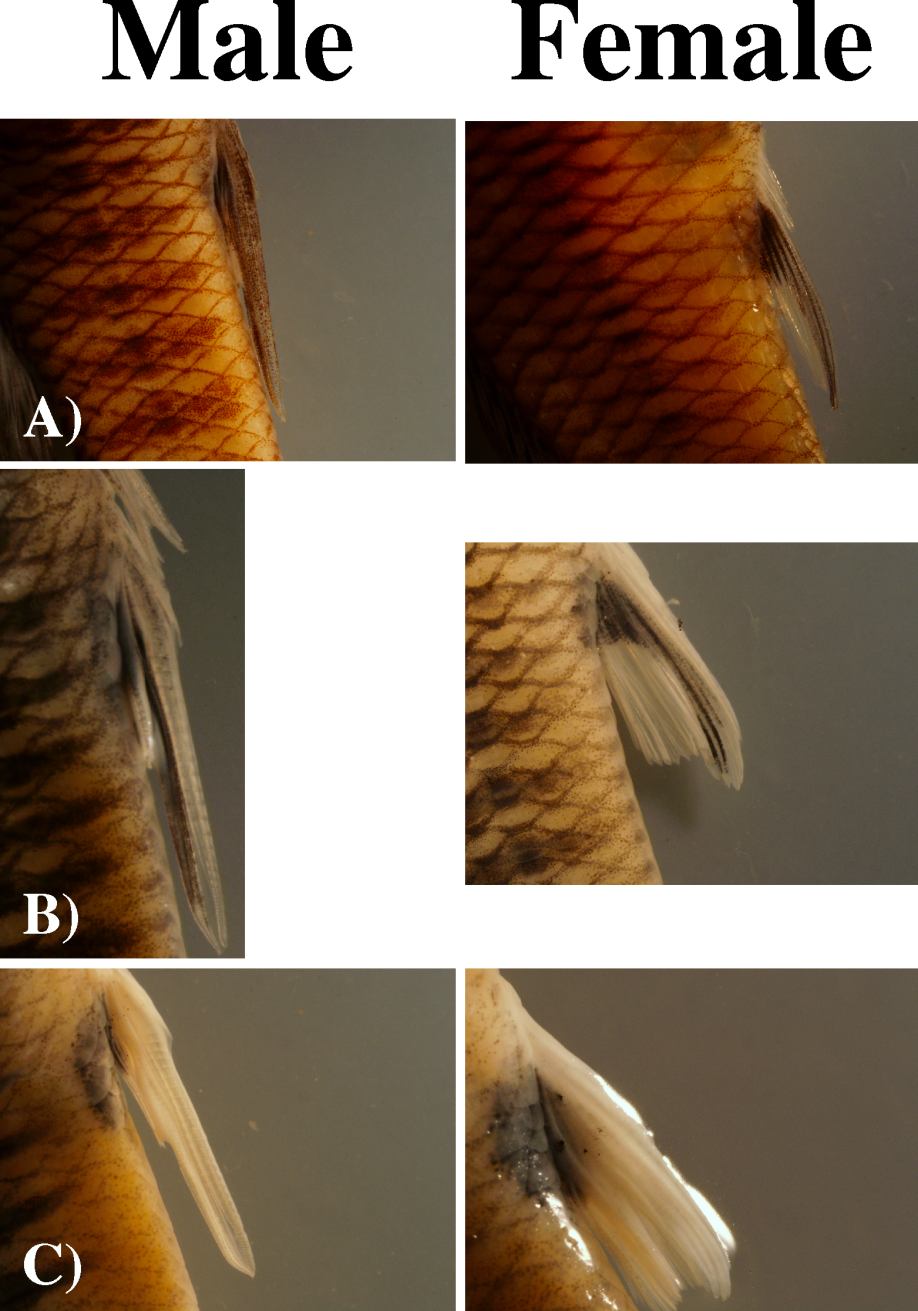
Gonopodial and anal fin pigmentation in male (on left) and female (on right). *Brachyrhaphis* fishes: (A) *B*. *rhabdophora*; (B) *B*. *roseni*; and (C) *B*. *terrabensis*.

We selected three species of *Brachyrhaphis* for this study: *B*. *terrabensis*, *B*. *roseni*, and *B*. *rhabdophora*. These species were chosen because each shows apparent mimicry in anal fin pigmentation between males and females, but the species vary from one another (Fig 1) [21]. Each species has a distinct form of gonopodial and anal fin pigmentation, although modest variation does exist among individuals within a species. *Brachyrhaphis terrabensis* males and females have little, and in some cases, no anal fin or gonopodial pigmentation; when pigmentation is present, it is expressed only as a small, circular area of pigment at the base of the fin. *Brachyrhaphis roseni* males have pigmentation that covers most of their gonopodium, while most females have a distinct, dark, gonopodial shape along their anal fin. *Brachyrhaphis rhabdophora* males have a heavily pigmented gonopodia, and all females have a dark, inverted triangle shape along their anal fin. We tested two populations of *B*. *rhabdophora* because prior work shows that a variety of traits have diverged within this species coincident with the presence or absence of predators (*Parachromis dovii*, Cichlidae) [28, 29]. Although there was no *a priori* reason to predict that predation would affect mimicry, we evaluated two populations of this species separately: ‘*B*. *rhabdophora* Javilla’ co-occurs with piscivorous predators and ‘*B*. *rhabdophora* Grande’ occurs in the absence of predators [30].

*Brachyrhaphis roseni* and *B*. *terrabensis* were collected from two streams in the Rio Caño Seco drainage in Puntarenas, Costa Rica in April 2014. *Brachyrhaphis roseni* were collected from an unnamed low-elevation tributary (N 8.65427, W 82.93489; elevation 70 m) and *B*. *terrabensis* collected from an unnamed high-elevation tributary (N 8.81299 W 82.97408; elevation 962 m). *Brachyrhaphis rhabdophora* populations were collected from two streams in Guanacaste Province, Costa Rica, in April 2014. One population was collected from low-elevation Rio Javilla (N 10.40245, W 85.07610; elevation 99 m) and the other population was collected from high-elevation Quebrada Grande (N 10.44194, W 84.98804; elevation 363 m). Lourdes Vargas Fallas and Javier Guevara of Sistema Nacional de Áreas de Conservación (SINAC) in Costa Rica approved all collecting and exporting permits.

After collection, fish were transported to Brigham Young University and were held in the lab in 5-gallon tanks. We covered the bottoms of each tank in small rocks and we added small plants to create a natural environment. Tanks were cleaned regularly. During the first week in the laboratory, we treated the fish for parasites. Fish were kept on a normal light schedule of 12 h day and 12 h night and temperature was kept at 21 degrees C. Fish were fed daily *ad libitum* with TetraMin® (Tetra, Melle, Germany). Upon completion of this study, we returned all fish to their holding tanks and kept them under this normal care practice. All fish utilized in this study were wild caught. This study was carried out in accordance with the recommendations in the Guide for the Care and Use of Laboratory Animals of the National Institutes of Health [31]. We followed a specific animal welfare protocol for this work, which was approved by the International Animal Care and Use Committee (IACUC) at Brigham Young University (Protocol number: 14–0601). Our sample sizes were based on the minimum number of individuals likely to yield a significant difference among treatments if such a difference actually existed. With input from the IACUC at BYU, and based on published work in other species, we attempted to run between 20 and 25 male choice test trials and between 3 and 5 female aggression trials.

### Experimental design

We designed our first experiment to determine if males were more likely to attend to non-pigmented females than to pigmented females with markings that mimic the male gonopodium. We designed our second experiment to determine how females interact in pairs, based on the anal fin pigmentation of each female in the pair. Females were placed in two types of pairs: one type with anal fin pigmentation on both females, or in pairs with only one female with anal fin pigmentation. We then totaled the number of aggressive displays shown by both females in a given pair. We also performed a test where we placed females in non-matching pigmentation pairs and counted the number of aggressive displays by individual females based on whether she was viewing a pigmented or non-pigmented female. All experiments were carried out between 08:00–14:00 over the course of two weeks.

#### Male attendance to females

We used a dichotomous choice test to evaluate male attendance behavior in *B*. *rhabdophora* Grande, *B*. *rhabdophora* Javilla, *B*. *roseni*, and *B*. *terrabensis*. If males spend more time associating with non-pigmented females than with pigmented females, this would be consistent with the hypothesis that sexual mimicry is used to deter unwanted male attention. We used association time as an indicator of attendance [32–34]. In order to control for differences in pigmentation patterns among wild-caught females, we used dry ice to freeze brand the anal fin, removing all pigment from each female prior to applying a pigment treatment [35]. To freeze brand the fin, we first anesthetized the fish via immersion using MS-222, then quickly, and carefully, touched the pigmented area with dry ice. We repeatedly touched the dry ice to the fin over a 30 second time period. We then placed the fish in a recovery tank and gave the fish 48 hours to recover. Similar methods have also been used to remove barring from swordtails (*Xiphophorus cortezi*) [36, 37]. These methods were effective at removing all pigment from the fin, and did not harm the fish in our study. Following pigment removal and recovery, we randomly marked half of the individuals with a temporary pigment (Dr. Naylor’s BLU-KOTE) in the shape of male gonopodium; the remaining individuals were left unmarked. To apply marks, we anesthetized each female with MS-222 and applied Dr. Naylor’s BLU-KOTE in the shape of the male gonopodium for each respective species; as a control, we anesthetized unmarked females and painted their anal fins with a wetted paintbrush.

We then tested male association time when males were given a choice between marked and unmarked females. We first grouped females into size-matched pairs, with the requirement that individuals in each pair had to be within 3 mm of each other. We then placed these two females, one painted and one control, in separate small tanks attached to either end of a larger central tank. We randomized the placement of the pigmented female (left or right) in each trial. The small side tanks measured 15 x 27 x 30.5 cm while the larger central tank measured 56 x 28 x 30.5 cm. Clear glass separated the central tanks from the side tanks. We housed these tanks in a soundproof room with full spectrum overhead lighting. We placed a male in the center of the central tank and recorded observations remotely outside of the experimental room via webcam. We allowed the male up to 10 minutes to acclimate to the tank. Once the male observed each female and returned to the center of the tank, we started a 10-minute observation period via webcam. A few males failed to interact with females during the acclimation period and simply held still at the bottom of the tank (*B*. *rhabdophora* Javilla n = 5; *B*. *rhabdophora* Grande n = 5; *B*. *roseni* n = 4; *B*. *terrabensis* n = 7). These trials were excluded from the study. We successfully tested 15 *B*. *rhabdophora* Grande males, 16 *B*. *rhabdophora* Javilla males, 19 *B*. *roseni* males, and 21 *B*. *terrabensis* males. We recorded data in the program ObjectTracker [38], which marks the location of the focal fish throughout the trial time.

We visually divided the central tank into three sections: two association zones on the ends, which were each 14 x 28 cm, and a center zone, which was 28 x 28 cm. We measured the amount of time (in seconds) each male spent in association zones with each treatment female. Time spent in the center zone was not included in the analysis as this space was considered neutral, inferring that the male had not “chosen” to associate with a female.

To ensure that Dr. Naylor’s BLU-KOTE was an appropriate substitute for melanin pigmentation in female anal fins, we used a dichotomous choice test as a control, giving males the choice between naturally pigmented and painted females. We included only *B*. *roseni* and *B*. *rhabdophora* populations in this control study, as there were no naturally pigmented female *B*. *terrabensis*. We analyzed time spent on each side of the tank using a Wilcoxon signed-rank test in the program JMP (SAS Institute). We found no statistical difference between time spent with naturally pigmented females versus females marked with Dr. Naylor’s BLU-KOTE (*B*. *rhabdophora* Grande: P = 0.89, n = 4; *B*. *rhabdophora* Javilla; P = 1.00, n = 4, *B*. *roseni*: P = 0.47, n = 4), suggesting that our pigment treatment was a valid surrogate.

#### Female dominance

To determine if females mimic male traits to signal dominance over other *Brachyrhaphis* females, we first compared the behavior of pairs of females that had similar levels of pigmentation to pairs of females that had different levels of pigmentation. Our first assay was to count the combined number of aggressive displays exhibited by two females that were placed together in a trial. In all trials, pairs of females were size-matched such that they did not differ by more than 3 mm in standard length. In the first treatment, females were matched for pigment levels. For *B*. *roseni* and *B*. *rhabdophora* populations, we utilized naturally pigmented females; for *B*. *terrabensis*, we applied Dr. Naylor’s BLU-KOTE in the shape of the anal fin pigmentation found on *B*. *rhabdophora* females (see above for a description of pigmentation patterns in each species). In the second treatment, we identified two size-matched females and used freeze branding to completely remove the pigmentation from both, after which they were given a 24-hour recovery period. Following this, we randomly chose one female to restore the pigment pattern using BLU-KOTE, while the second female was painted with a wet paintbrush as a control.

The behavior trials were conducted by placing pairs of females in a small tank (26.7 x 15.25 x 17 cm) and observing aggressive behaviors via webcam from a separate room. Females were given up to 45 minutes to acclimate and establish dominance. Once either female showed an aggressive signal (nips, chases, or “S” displays) we recorded behavior for 15 consecutive minutes. Nips were scored when an individual bit or tried to bite her partner. Chases were scored when a female quickly swam towards the other female. “S” displays were scored when a female arched her spine in an ‘s’ shape with fins erect and presented herself to her counterpart [23]. The number of aggressive displays were totaled for each female. We also combined the total number of aggressive displays demonstrated by both females in a pair.

The purpose of our second assay was to look specifically at the number of aggressive displays of an individual female based on the pigmentation pattern of the female with which she interacted. Females that had not been used in previous tests were placed in size-matched pairs (within 3 mm) and freeze branded to remove all anal fin pigmentation. After a 24-hour recovery period we added gonopodial pigmentation to one female using BLU-KOTE and the second female was painted with a wetted brush as a control. Similar to the first assay, we placed this non-matching pair in a small tank and gave females up to 45 minutes to acclimate and show an aggressive display. After the first aggressive display we recorded behavior for 15 consecutive minutes. For this assay, instead of measuring the total number of aggressive displays, we counted the number of displays shown by each individual female based on the pigmentation of the opposing female.

### Statistical analyses

We employed non-parametric statistical approaches for all of our tests given that our data failed to meet the assumptions of parametric statistics. To evaluate male association times, we used a Wilcoxon signed-rank test to compare the amount of time males of each species spent with pigmented versus non-pigmented females. To analyze tests of female aggressive displays, we compared total number of aggressive behaviors in a trial (sum of both females) between naturally pigmented pairs and non-matching pigmented/non-pigmented pairs. These count data were compared using Mann-Whitney U test. We also analyzed the number of aggressive behaviors displayed by individual females in a non-matched pair using a Wilcoxon signed-rank test. All data were analyzed using JMP (SAS Institute).

## Results

**Fig 2:**
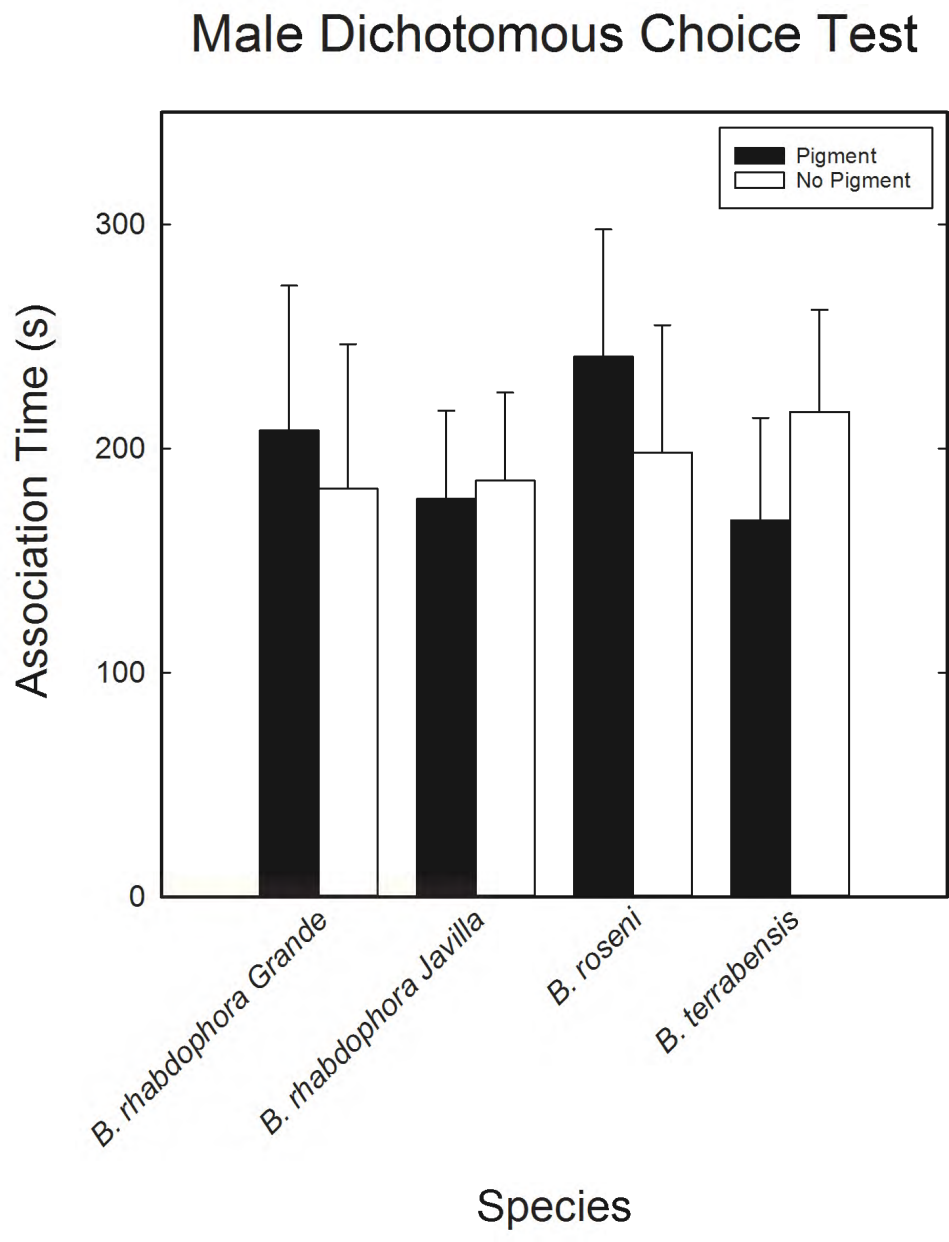
Results of male dichotomous choice test comparing the amount of time (in seconds) males spent with either pigmented or non-pigmented females. In all species the difference in the association time between pigmented and non-pigmented females was not statistically significant (P>0.05 in all cases). Error bars represent standard error calculations.

In each of the three *Brachyrhaphis* species, we found no difference in the amount of time males spent associating with either pigmented or non-pigmented females (Fig 2). Moreover, males from both *B*. *rhabdophora* populations were almost equal in the amount of time spent with pigmented and non-pigmented females (S1 Table).

**Fig 3:**
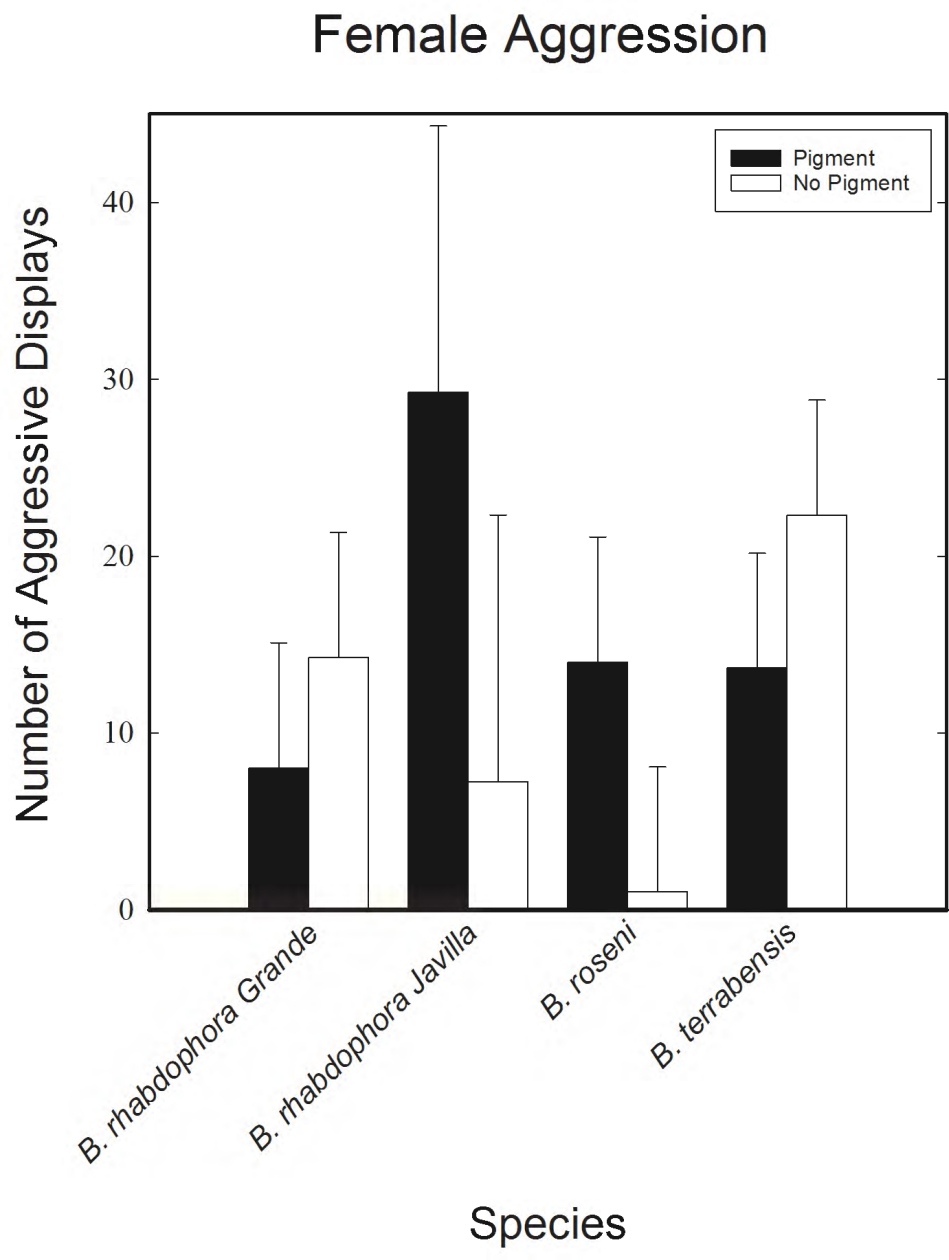
Female aggression. Number of aggressive events by an individual female based on what type of female she interacted with (a pigmented or non-pigmented female). In all species the difference in aggressive displays between pigmented and nonpigmented females was not statistically significant (P>0.05 in all cases).Error bars represent standard error calculations.

In *B*. *roseni*, *B*. *rhabdophora* Grande, and *B*. *rhabdophora* Javilla females showed no statistically significant difference in aggression levels in the first assay between female pairs with matching pigmentation versus female pairs with non-matching pigmentation (our first assay described above). In *B*. *terrabensis*, non-matching pigment females did show a higher number of aggressive displays than matching pigment female pairs (see Table 1 for analysis, see S2 Table for data). Finally, in all species we found no statistical differences in the number of aggressive events between females observing a pigmented counterpart and females observing a non-pigmented counterpart (Fig 3 and S2 Table).

**Table 1:** A comparison of aggression levels between pairs of females with either matching or non-matching levels of anal fin pigmentation evaluated by species.

SPECIES	PAIR TYPE	NUMBER OF PAIRS	SCORE SUM	EXPECTED	MEAN	P-VALUE
***B*. *rhabdophora* Grande**	Non-Matching	4	22	18	5.50	0.20
	Matching	4	14	18	3.50	
***B*. *rhabdophora* Javilla**	Non-Matching	4	22	18	5.89	0.14
	Matching	4	14	18	3.13	
***B*. *roseni***	Non-Matching	3	22	18	4.50	0.19
	Matching	3	14	18	2.50	
***B*. *terrabensis***	Non-Matching	3	17.5	12	5.83	0.02*
	Matching	4	10.5	16	2.63	

Females were either both naturally pigmented or one pigmented and one with pigment removed. In the case of *B*. *terrabensis*, females in “same” pairs were pigmented with BLU-KOTE. The “score sum” refers to the sum of the rank score for each level. The “expected” is the expected score under the null hypothesis that there is no difference among levels. The “mean” shows the score mean of each sample.

* Indicates a significant difference between treatments.

## Discussion

We found no evidence of adaptive female sexual mimicry of males in *Brachyrhaphis* fishes. Our results were consistent across all three species examined here. Unlike other systems where male-like pigmentation in females is hypothesized to reduce male harassment [8, 17, 18], the presence of gonopodial pigmentation on female anal fins in our fishes had no effect on male association behavior. This stands in contrast to other systems, such as capuchin birds (*Perrisocephalus tricolor*), where males and females are sufficiently monomorphic that, at a distance, males cannot distinguish between sexes and sometimes must wait until females reveal their sex in order to identify potential mates [39]. *Brachyrhaphis* males can apparently detect females at the close distances examined in our study, even those with gonopodial markings in their anal fins. One interesting possibility would be to run our experiments at greater distances to see if males would be more likely to be deceived by females with male gonopodial markings.

We also found no evidence that pigmentation of female anal fins was used in establishing dominance hierarchies in *Brachyrhaphis* females. Female fish interacting with non-pigmented females did not show higher levels of aggression than females interacting with pigmented counterparts. Yong et al. [40] explored a similar question in three-spined sticklebacks, and found no difference in aggression levels between females that had red throat patches versus those that do not. These findings are consistent with a growing list of studies, which show that in a variety of species, females ornamented with male sexual traits do not affect female aggression behavior (see [41] for a meta-analysis).

We evaluated females in pairs where both individuals were pigmented, and also in mismatched pairs, where we artificially removed pigment from both females and then applied artificial pigment to one. We found no difference in aggression between the pigmented pairs and the non-matched pigmented pairs, with one exception: *Brachyrhaphis terrabensis* females in non-matching pigmented pairs had higher levels of aggression than the matched pigmented pairs. This was opposite of what we predicted. The most likely explanation for this is that *Brachyrhaphis terrabensis* females almost completely lack natural pigmentation in the wild. Hence, our manipulation of adding pigment to one individual could have elicited increased levels of aggression if she was simply perceived as being unfamiliar or another species. Examples of organisms showing higher levels of aggression when interacting with an unfamiliar individual are well known (e.g. cichlid fishes, [42]).

Given that neither of the hypotheses evaluated here explains female anal fin pigmentation in *Brachyrhaphis* fishes, what other explanations exist? It is possible that anal fin and gonopodial pigmentation could be used as a species recognition cue. Although *Brachyrhaphis* species almost never occur in sympatry, they sometimes occur with a suite of other poeciliid fishes (including *Poecilia*, *Poeciliopsis*, and *Priapichthys* [43]). It is also possible that the shared pigmentation between males and females is simply non-adaptive. Lande [44] suggested that the presence of male traits in females, in some systems, is often non-adaptive and simply reflects cases where dimorphisms have not yet evolved. For example, in caribou, males typically have large antlers used in sexual displays, whereas females have small antlers similarly structured to males but apparently not used in any signaling context [45]. Yet antlers in females remain, simply because shared alleles are expressed in both sexes [46]. In a similar manner, the evolutionary benefit of female anal fin pigmentation in *Brachyrhaphis* fishes currently lacks conclusive data to provide an adequate explanation, consistent with the possibility that the trait is non-adaptive. Evidence for the genetic similarity of this trait could be found by identifying alleles that code for anal fin pigmentation and comparing the genetic similarity between males and females. In summary, what ultimately causes the striking pattern of anal fin mimicry in *Brachyrhaphis* fishes remains unknown, but adaptive explanations relating to harassment avoidance and dominance hierarchies are simply not supported by the data.

## Supporting information

S1 Table Male mate choice data. (XLSX)

S2 Table Female aggression data. (XLSX)

S1 Text ARRIVE checklist. (PDF)
